# Treatment of Broncho-Pneumonia in Children by Subcutaneous Injections of the Hydro-Chlorate of Quinine

**Published:** 1891-12

**Authors:** 


### Selections and Abstracts

### TREATMENT OF BRONCHO-PNEUMONIA IN CHILDREN BY SUBCUTANEOUS INJECTIONS OF THE HYDRO-CHLORATE OF QUININE.

In the *Journal de Medecene de Paris*, June 21, 1891, Dr. St.-Philippe calls attention to the difficulty in diagnosis between pneumonia complicated with enteritis and typhoid fever, while from the point of view of treatment he points out that there are two features which are valuable indications as to the methods to be pursued. First, as to the degree of bronchitis; and second, as to the extent of the pulmonary lesion, for the congestive process may be so sudden and expensive as to produce death in a few hours. Against this latter form of danger Dr. St.-Philippe maintains that there is no drug which can equal quinine, whether administered by the mouth, the rectum or under the skin. Sulphate of quinine he gives in black coffee, while in very small children of those who refuse to take medicine it may be employed in enemata or in powders; but both these modes of medication are slow in action, and for entire reliability he usually recommends the employment of quinine in subcutaneous injections, making use of a solution of chlorydrate of quinine and equal parts of glycerin and water. Quinine thus forms his main reliance in the treatment of roncho-pneumonia, to which may be added blisters, which he even allows to pass on to suppuration over the localities where rales are most abundant. In suffocative catarrh he has recourse to mustard plasters, large blisters or leeches, according to the age of the child; while in some cases he employs tincture of aconite root in doses of 5 to 20 drops in twenty-four hours, and in syrup of aconite in cases where the bronchitis is intense, while quinine and punch are given when the general state is at all depressed. In states of excitement he does not advise the use of opium, but warm baths and small doses of antipyrin. In severe cases, quinine and aconite are to be abandoned and caffeine given subcutaneously, and reliance placed upon digitalis and alcohol as stimulants. He likewise states that oxygen inhalations have given better results than injections of ether.—*Therapeutic Gazette.*

